# Inter‐alpha‐trypsin inhibitor heavy chain 4: A serologic marker relating to disease risk, activity, and treatment outcomes of rheumatoid arthritis

**DOI:** 10.1002/jcla.24231

**Published:** 2022-01-22

**Authors:** Kejian He, Sanshan He, Min Su

**Affiliations:** ^1^ Department of Rheumatology The First College of Clinical Medical Science Three Gorges University & Yichang Central People’s Hospital Yichang China; ^2^ Department of Rheumatism Immunology Minda Hospital of Hubei Minzu University Enshi China; ^3^ Department of Rheumatology and Immunology The People’s Hospital of China Three Gorges University The First People’s Hospital of Yichang The Institute of Autoimmune Disease of China Three Gorges University Hubei China

**Keywords:** clinical features, inflammatory cytokines, inter‐alpha‐trypsin inhibitor heavy chain 4, rheumatoid arthritis, treatment outcomes

## Abstract

**Objective:**

Inter‐alpha‐trypsin inhibitor heavy chain 4 (ITIH4) regulates immunity and inflammation, but its clinical role in rheumatoid arthritis (RA) patients remains unclear. Hence, this study was conducted to explore the association of circulating ITIH4 with disease risk, clinical features, inflammatory cytokines, and treatment outcomes of RA.

**Methods:**

After the enrollment of 93 active RA patients and 50 health controls (HCs), their serum ITIH4 level was analyzed by enzyme‐linked immunosorbent assay (ELISA). For RA patients only, serum ITIH4 level at week (W) 6 and W12 after treatment was also analyzed. Besides, serum tumor necrosis factor‐alpha (TNF‐α), interleukin (IL)‐1β, IL‐6, and IL‐17A at baseline of RA patients were also detected by ELISA.

**Results:**

ITIH4 was downregulated in RA patients (151.1 (interquartile range (IQR): 106.2–213.5) ng/mL) than in HCs (306.8 (IQR: 238.9–435.1) ng/mL) (*p *< 0.001). Furthermore, ITIH4 was negatively related to C‐reactive protein (CRP) (*r_s_* = −0.358, *p *< 0.001) and 28‐joint disease activity score using erythrocyte sedimentation rate (DAS28‐ESR) (*r_s_* = −0.253, *p *= 0.014) in RA patients, but not correlated with other clinical features (all *p *> 0.05). Besides, ITIH4 was negatively linked with TNF‐α (*r_s_* = −0.337, *p *= 0.001), IL‐6 (*r_s_* = −0.221, *p *= 0.033), and IL‐17A (*r_s_* = −0.368, *p* < 0.001) in RA patients, but not correlated with IL‐1β (*r_s_* = −0.195, *p* = 0.061). Moreover, ITIH4 was gradually elevated in RA patients from baseline to W12 after treatment (*p* < 0.001). Additionally, the increment of ITIH4 at W6 and W12 was linked with treatment response and remission in RA patients (all *p* < 0.05).

**Conclusion:**

Circulating ITIH4 possesses clinical utility in monitoring disease risk, inflammation, disease activity, and treatment outcomes of RA.

## Graphical abstract

A total of 93 active RA patients and 50 HCs were enrolled in this study; then, their serum ITIH4 was detected by ELISA. For RA patients only, their serum ITIH4 at W6 and W12, as well as serum TNF‐α, IL‐1β, IL‐6, and IL‐17A at baseline were also detected by ELISA. After that, we obtained some interesting findings. ITIH4 was downregulated in RA patients than HCs. Besides, ITIH4 negatively correlated with CRP, DAS28‐ESR, TNF‐α, IL‐6, and IL‐17A in RA patient. Moreover, ITIH4 was gradually elevated in RA patients during the treatment; meanwhile, the increment of ITIH4 at W6 and W12 was linked with treatment response and remission in RA patients.

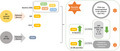

## 1 INTRODUCTION

Rheumatoid arthritis (RA) is an autoimmune disease characterized by the accumulation of synovial hyperproliferation and inflammation.^1^, ^2^, ^3^, ^4^ Moreover, the immune mediate inflammation might further erode articular bone and lead to joint deformity, bone destruction, and disability.^1^, ^2^, ^3^, ^4^, ^5^ In addition to severe symptoms described above, excess comorbidities (including fragility fracture, osteoarthritis, etc) are also along with RA patients, which could reduce their quality of life.^6^, ^7^ Aiming to control symptoms and progression of RA, many treatments have been applied, which mainly include nonsteroidal anti‐inflammatory drug (NSAID), conventional synthetic disease‐modifying antirheumatic drugs (cDMARDs), glucocorticoids (GC), biologic DMARDs, etc.^8^, ^9^, ^10^, ^11^ However, many RA patients still suffer from poor treatment response and remission; therefore, exploring biomarkers assisting to predict treatment outcomes can help the clinicians to stratify RA patients, individualize their treatment, and improve the outcomes in RA patients.^12^, ^13^

Inter‐alpha inhibitor proteins (IAIPs), a family of serine proteases inhibitors, comprise of inter‐alpha inhibitor (2 heavy chains and 1 light chain) and pre‐alpha inhibitor (1 heavy chain and 1 light chain).^14^, ^15^ Several studies disclose the anti‐inflammatory properties of IAIPs in some inflammation‐implicated diseases, such as sepsis, enterocolitis, stroke, recurrent pregnancy loss, and allergic contact dermatitis^16^, ^17^, ^18^, ^19^, ^20^ In terms of inter‐alpha‐trypsin inhibitor heavy chain 4 (ITIH4), a plasma glycoprotein produced by liver, belongs to the family of IAIPs.^21^, ^22^ Like other IAIP family members, ITIH4 also has systemic anti‐inflammatory properties in some complex diseases, including Alzheimer's disease, acute ischemic stroke, etc.^23^, ^24^ With regard to RA, previous studies find that citrullinated form of ITIH4 is differentially expressed in joints of RA patients and it fluctuates with disease activity score, which indicates that citrullinated ITIH4 may participate in the inflammation response of RA.^25^, ^26^, ^27^ However, the role of circulating ITIH4 level in clinical management of RA patients has not been examined yet. In our preliminary study with a relatively small sample size, we observed a decrement of ITIH4 in RA patients compared with controls.

Hence, this study detected serum ITIH4 in RA patients (before and after treatment) and health controls (HCs), in order to explore the association of circulating ITIH4 with disease risk, clinical features, inflammatory cytokines, and treatment outcomes of RA.

## 2 MATERIALS AND METHODS

### 2.1 Subjects

Between July 2018 and April 2021, 93 active RA patients were consecutively recruited in this study. Eligible patients were ≥18 years with a diagnosis of RA and fulfilled the 2010 American College of Rheumatology/European League Against Rheumatism Rheumatoid criteria.^28^ All patients had active RA, defined as 28‐joint disease activity score using erythrocyte sedimentation rate (DAS28‐ESR) > 3.2 at screening. The exclusion criteria included serious infections within 6 months before enrollment, hematological or autoimmune diseases, severe liver and kidney diseases, malignancies, or a history of cancer. The HCs group was formed by a total of 50 healthy individuals who were gender (male vs. female, 1:4) and age (40–70 years) matched with RA patients. The exclusion criteria for HCs were consistent with those for RA patients; besides, subjects with immune‐related diseases were also excluded. The Ethics Committee approved the study, and written informed consent was obtained from each subject.

### 2.2 Data recording

Demographic data, medication histories, laboratory tests, and physical examinations were recorded at enrollment. Laboratory tests included C‐reactive protein (CRP), erythrocyte sedimentation rate (ESR), anti‐citrullinated protein antibodies (ACPA), and rheumatoid factor (RF). Physical examinations included tender joint count (TJC), swollen joint count (SJC), and Health Assessment Questionnaire Disability Index (HAQ‐DI) score. The DAS28‐ESR was calculated by the formula without assessment of general health according to a previous study.^29^

### 2.3 Sample collection

Blood samples were taken from RA patients at baseline (before treatment), week 6 (W6), and week 12 (W12) after treatment. Meanwhile, blood samples of HCs were also taken after enrollment. All samples were centrifuged (1500 g, 10 min, 25℃) to separate the serums for further detection. The serums of RA patients and HCs were used to detect the level of ITIH4. Moreover, the serum levels of tumor necrosis factor‐alpha (TNF‐α), interleukin‐1 beta (IL‐1β), interleukin‐6 (IL‐6), and interleukin‐17A (IL‐17A) of RA patients at baseline were also measured.

### 2.4 Quantification of Cytokines and ITIH4 in Serum

The ITIH4 in serum was analyzed by enzyme‐linked immunosorbent assay (ELISA) using Human ITIH4 DuoSet ELISA (DY8157‐05; R&D, Minneapolis, Minnesota, USA). The TNF‐α, IL‐1β, IL‐6, and IL‐17A in serum were detected using commercial ELISA kits (R&D, Minneapolis, Minnesota, USA) containing antibodies raised against target cytokines. The tests were performed strictly according to the manufacturer's instructions. The brief steps were as follows: 100 μL of sample or standards were added in diluent per well and incubated for 2 h at 24℃. Each well was aspirated and washed for 3 times. Then, 100 μL of the diluted detection antibody was added to every well and incubated for 2 h at 24℃, followed by repeated washing for 3 times. Following that, 100 µL of substrate solution was added to every well and incubated for 20 min at 24℃ avoiding direct light. After that, 50 μL of stop solution was added to each well. Finally, determination of the optical density of each well was immediately performed by a microplate reader set to 450 nm.

### 2.5 Treatment

Considering disease situation, doctor's advice, and patients’ wishes, some patients chose biologics‐based regimen including tumor necrosis factor inhibitor (eg, etanercept 25 mg twice a week, subcutaneous injection) or interleukin‐6 inhibitor (eg, tocilizumab 8 mg/kg, once every 4 weeks intravenously), while others received monotherapy or combination therapy of conventional disease‐modifying antirheumatic drugs (cDMARDs) including methotrexate (7.5~20 mg once a week orally), sulfasalazine (2~3 g three times a day orally), and leflunomide (20 mg once a day orally).

### 2.6 Follow‐up and evaluation

Patients were followed up at W6 and W12 after treatment. Two patients were lost to follow‐up within W6, and another five were lost to follow‐up within W12, resulting in a total of seven patients lost to follow‐up. The clinical response and remission were evaluated at W12. The clinical response was defined as >1.2 decline of DAS28‐ESR from baseline,^30^ and the clinical remission was defined as DAS28‐ESR <2.6 points.^31^ Based on the clinical response and remission at W12, patients were classified as response patients, non‐response patients, remission patients, and non‐ remission patients, accordingly.

### 2.7 Statistical analyses

SPSS 26.0 (IBM Corp., Armonk, New York, USA) was used for statistical analyses. Graphs were plotted by GraphPad Prism 7.01 (GraphPad Software Inc., San Diego, California, USA). Difference analyses of ITIH4 in different groups were analyzed by Mann‐Whitney U test, and the ability of ITIH4 in identifying RA patients from HCs was estimated by receiver operating characteristic (ROC) curve analysis. Mann‐Whitney U test and Spearman's rank correlation coefficient were used to analyze the correlations between inflammatory cytokines, clinical futures, treatments, and ITIH4. The change in ITIH4 from W0 (baseline) to W12 was analyzed by the Friedman test. All tests were two‐sided, and *p* < 0.05 was regarded as statistical significance. Multivariate logistic regression analysis with forward stepwise method was conducted to analyze the potential factors affecting clinical response at W12 in RA patients.

## 3 RESULTS

### 3.1 Characteristics of RA patients

The mean age of RA patients in this study was 56.1 ± 9.6 years with 21 (22.6%) males and 72 (77.4%) females (Table 1). In terms of their clinical and serological parameters, the median disease duration of them was 2.7 (1.1–5.1) years; moreover, there were 19 (20.4%) RF‐negative patients and 74 (79.6%) RF‐positive patients; 38 (40.9%) ACPA‐negative patients and 55 (59.1%) ACPA‐positive patients in this study. Furthermore, the mean DAS28‐ESR of RA patients was 5.1 ± 0.7. The detailed characteristics of RA patients were listed in Table 1.

**TABLE 1:** Characteristics of RA patients

Items	RA patients (N = 93)
Demographics	
Age (years), mean ± SD	56.1 ± 9.6
Gender, n (%)	
Male	21 (22.6)
Female	72 (77.4)
BMI (kg/m^2^), mean±SD	23.0 ± 3.1
Clinical and serological parameters	
Disease duration (years), median (IQR)	2.7 (1.1–5.1)
RF, n (%)	
Negative	19 (20.4)
Positive	74 (79.6)
ACPA, n (%)	
Negative	38 (40.9)
Positive	55 (59.1)
TJC, median (IQR)	7.0 (4.0–9.5)
SJC, median (IQR)	6.0 (3.5–9.0)
ESR (mm/h), mean ± SD	35.4 ± 16.3
CRP (mg/L), median (IQR)	25.5 (14.3–38.6)
DAS28‐ESR, mean ± SD	5.1 ± 0.7
HAQ‐DI score, mean ± SD	1.2 ± 0.3

Abbreviations: RA, rheumatoid arthritis; SD, standard deviation; BMI, body mass index; IQR, interquartile range; RF, rheumatoid factor; ACPA, anti‐citrullinated protein autoantibody; TJC, tender joint count; SJC, swollen joint count; ESR, erythrocyte sedimentation rate; CRP, C‐reactive protein; DAS28, 28‐joint Disease Activity; HAQ‐DI, Health Assessment Questionnaire Disability Index.

### 3.2 Level of ITIH4 in RA patients and HCs

**FIGURE 1:**
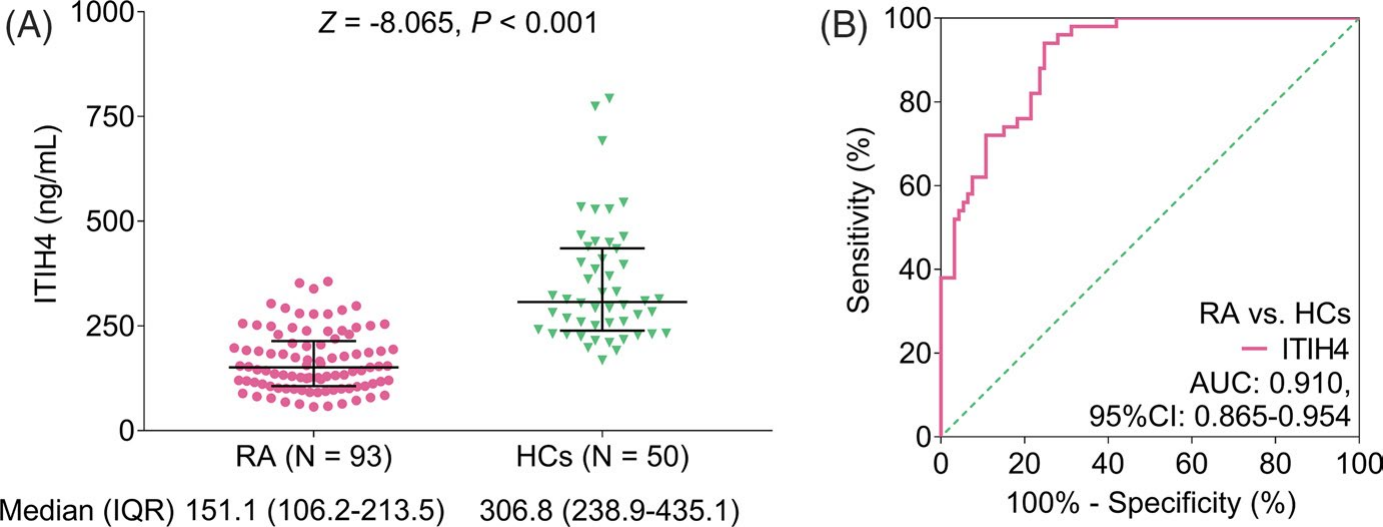
ITIH4 was downregulated in RA patients compared than HCs. Comparison of ITIH4 level between RA patients and HCs (A); and diagnostic performance of ITIH4 to distinguish RA patients from HCs (B)

ITIH4 level was declined in RA patients compared with HCs (*p *< 0.001, Figure 1A); meanwhile, median ITIH4 of RA patients and HCs was 151.1 (interquartile range (IQR): 106.2–213.5) ng/mL and 306.8 (IQR: 238.9–435.1) ng/mL, respectively. Besides, ROC curve disclosed that ITIH4 had good diagnostic value to distinguish RA patients from HCs (area under the curve (AUC): 0.910, 95% confidence interval (CI): 0.865–0.954); furthermore, the sensitivity and specificity were 0.940 and 0.753, respectively, at the best cutoff point which was screened out from the values giving the maximum sum of specificity and sensitivity (Figure 1B).

### 3.3 Correlation of ITIH4 with clinical features and inflammatory cytokines in RA patients

**FIGURE 2:**
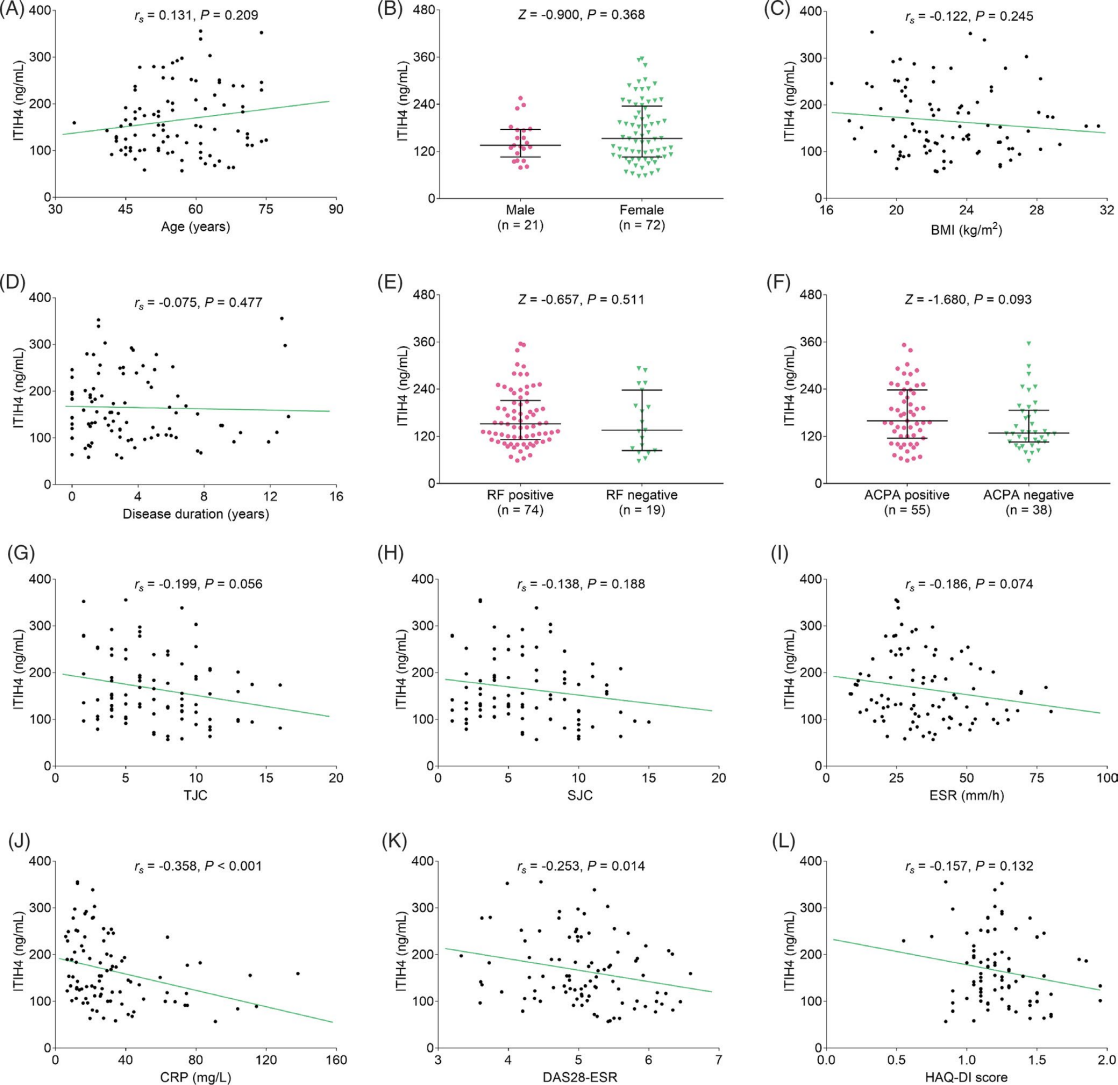
ITIH4 was negatively correlated with CRP and DAS28‐ESR in RA patients. Correlation of ITIH4 with age (A), gender (B), BMI (C), disease duration (D), RF (E), ACPA (F), TJC (G), SJC (H), ESR (I), CRP (J), DAS28‐ESR (K), and HAQ‐DI (L) in RA patients

In RA patients, ITIH4 was negatively related to CRP (*r_s_* = −0.358, *p* < 0.001) and DAS28‐ESR (*r_s_* = −0.253, *p* = 0.014), while ITIH4 was not correlated with age (*r_s_* = 0.131, *p* = 0.209), gender (*Z* = −0.900, *p* = 0.368), body mass index (BMI) (*r_s_* = −0.122, *p* = 0.245), disease duration (*r_s_* = −0.075, *p* = 0.477), RF (*Z* = −0.657, *p* = 0.511), ACPA (*Z* = −1.680, *p* = 0.093), TJC (*r_s_* = −0.199, *p* = 0.056), SJC (*r_s_* = −0.138, *p* = 0.188), ESR (*r_s_* = −0.186, *p* = 0.074), or HAQ‐DI score (*r_s_* = −0.157, *p* = 0.132) (Figure 2A‐L).

**FIGURE 3:**
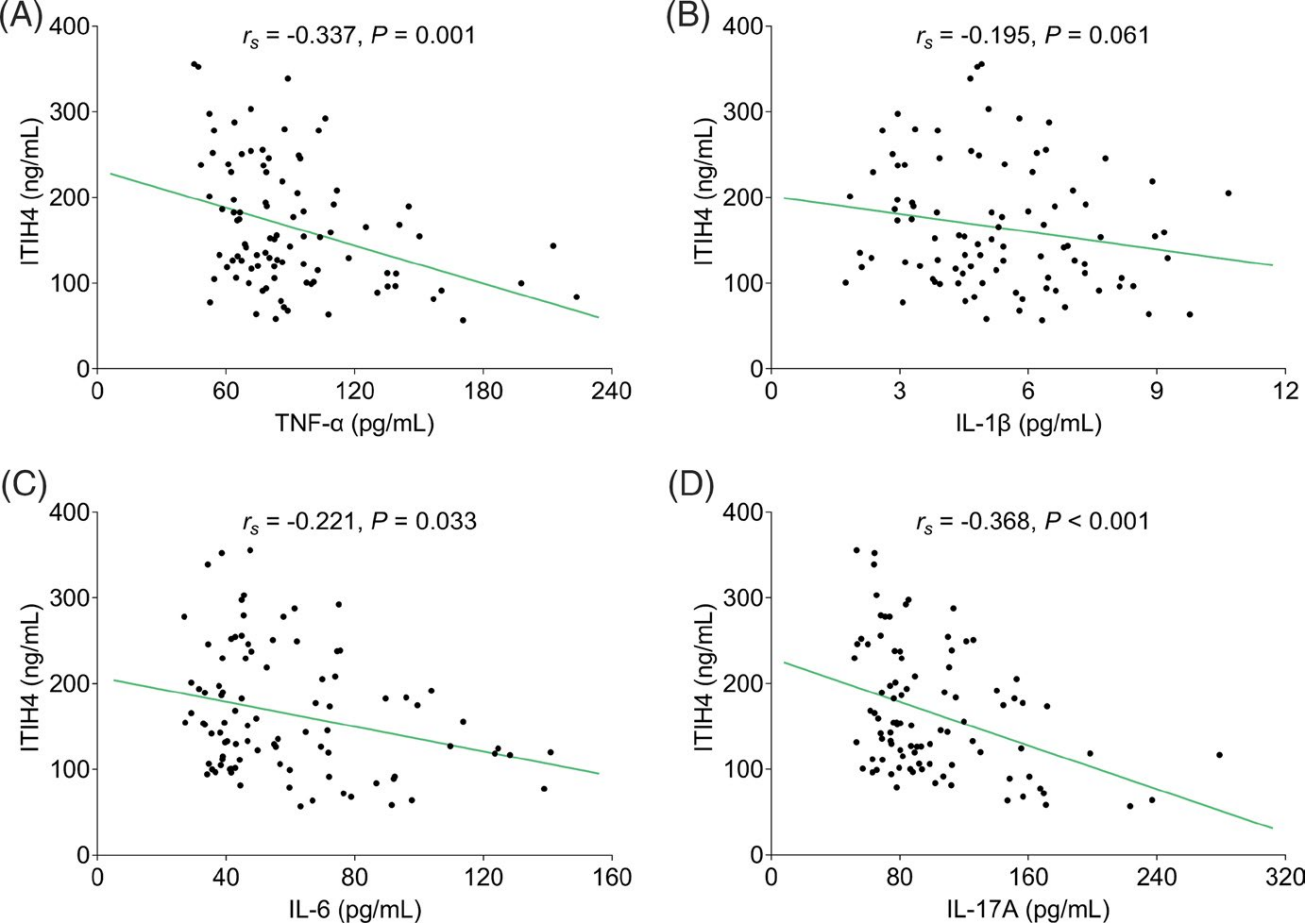
ITIH4 was negatively correlated with TNF‐α, IL‐6, and IL‐17A in RA patients. Correlation of ITIH4 with TNF‐α (A), IL‐1β (B), IL‐6 (C), and IL‐17A (D) in RA patients

In terms of inflammatory cytokines, ITIH4 was negatively linked with TNF‐α (*r_s_* = −0.337, *p* = 0.001), IL‐6 (*r_s_* = −0.221, *p* = 0.033), and IL‐17A (*r_s_* = −0.368, *p* < 0.001) in RA patients, but not correlated with IL‐1β (*r_s_* = −0.195, *p* = 0.061) (Figure 3A‐D).

### 3.4 The relationship between ITIH4 and the treatment regimens

With regard to the treatment history of RA patients, a total of 58 (62.4%) patients received NSAID; 79 (84.9%) patients were treated with GC; 79 (84.9%) patients received cDMARD; and 28 (30.1%) patients were treated with biologics (Table 2). As to current treatment, 72 (77.4%) patients received cDMARD and 21 (22.6%) patients were treated with biologics‐based regimen.

**TABLE 2:** Treatments of RA patients

Items	RA patients (N = 93)
History of treatment	
NSAID, n (%)	
No	35 (37.6)
Yes	58 (62.4)
GC, n (%)	
No	14 (15.1)
Yes	79 (84.9)
cDMARD, n (%)	
No	14 (15.1)
Yes	79 (84.9)
Biologics, n (%)	
No	65 (69.9)
Yes	28 (30.1)
Current treatment	
cDMARD (Monotherapy or combination), n (%)	
No	21 (22.6)
Yes	72 (77.4)
Biologics‐based regimen (TNFi or IL−6i), n (%)	
No	72 (77.4)
Yes	21 (22.6)

Abbreviations: RA, rheumatoid arthritis; NSAID, nonsteroidal anti‐inflammatory drug; GC, glucocorticoid; cDMARD, conventional disease‐modifying antirheumatic drug; TNFi, tumor necrosis factor inhibitor; IL‐6i, interleukin‐6 inhibitor.

In terms of the association of ITIH4 with treatments, there was no correlation of ITIH4 with history of treatment or current treatment regimens in RA patients (all *p* > 0.050) (Table 3).

**TABLE 3:** Correlation of ITIH4 expression with treatments in RA patients

Items	ITIH4 (ng/mL)^*^, median (IQR)	*Z*	*P* value
History of treatment			
NSAID		−0.056	0.956
No	143.5 (111.1–208.3)		
Yes	153.0 (101.4–237.6)		
GC		−0.150	0.880
No	163.1 (115.0–205.4)		
Yes	151.1 (106.0–218.7)		
cDMARD		−0.150	0.880
No	163.1 (115.0–205.4)		
Yes	151.1 (106.0–218.7)		
Biologics		−0.637	0.524
No	151.1 (105.5–203.3)		
Yes	149.9 (112.7–248.4)		
Current treatment			
cDMARD (Monotherapy or combination)		−0.524	0.600
No	151.1 (105.5–184.5)		
Yes	147.9 (111.3–235.5)		
Biologics‐based regimen (TNFi or IL−6i)		−0.524	0.600
No	147.9 (111.3–235.5)		
Yes	151.1 (105.5–184.5)		

Abbreviations: ITIH4, inter‐alpha‐trypsin inhibitor heavy chain 4; IQR, interquartile range; NSAID, nonsteroidal anti‐inflammatory drug; GC, glucocorticoid; cDMARD, conventional disease‐modifying antirheumatic drug; TNFi, tumor necrosis factor inhibitor; IL‐6i, Interleukin‐6 inhibitor. *, ITIH4 level at baseline.

### 3.5 Association of ITIH4 with treatment outcomes in RA patients

**FIGURE 4:**
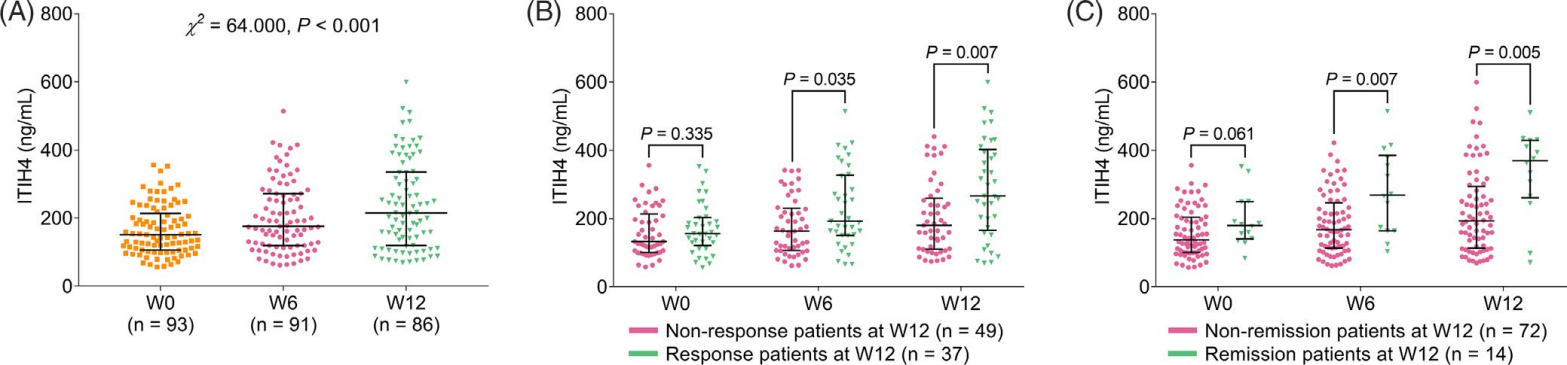
ITIH4 was gradually increased during 12‐week treatment in RA patients and correlated with treatment response and remission. Comparison of ITIH4 at different timepoints in RA patients (A); and the correlation of ITIH4 with treatment response (B) as well as treatment remission (C) in RA patients

ITIH4 was gradually elevated in RA patients from baseline to W12 after treatment (*p* < 0.001, Figure 4A). Moreover, ITIH4 at W0 was not correlated with treatment response (*p* = 0.335), while higher ITIH4 at W6 (*p* = 0.035) and W12 (*p* = 0.007) was related to treatment response in RA patients (Figure 4B). Besides, ITIH4 at W0 was not linked with treatment remission (*p* = 0.061), while higher ITIH4 at W6 (*p* = 0.007) and W12 (*p* = 0.005) was associated with treatment remission in RA patients (Figure 4C).

### 3.6 Changes of ITIH4 in patients receiving cDMARDs or biologics

ITIH4 was of no difference in RA patients with different current treatments (cDMARD vs. biologics) (*p* = 0.600, Supplementary figure S1A), while ITIH4 was gradually elevated from baseline to W12 after treatment in patients treated with cDMARD (*p* < 0.001, Supplementary figure S1B). Additionally, ITIH4 was gradually increased from baseline to W12 after treatment in patients treated with biologics‐based regimen (*p* = 0.035, Supplementary figure S1C).

Independent predictive factors of clinical response at W12 in RA patients.

Multivariate logistic regression model was applied to further analyze the independent factors for clinical response at W12 in RA patients, which indicated that higher ITIH4 at W12 (*p *= 0.001) and higher CRP (*p *= 0.005) were independent factors for clinical response at W12 in RA patients (Supplementary table S1).

## 4 DISCUSSION

ITIH4 is known as a negative acute‐phase inflammatory response protein, which belongs to IAIPs family and protects against the damaging effects of several proteases.^16^, ^21^, ^32^ Previous studies have determined the abnormal level of ITIH4 in different diseases, including chronic obstructive pulmonary disease (COPD), recurrent pregnancy loss, acute ischemic stroke, etc.^19^, ^33^ For example, one study finds that ITIH4 is downregulated in COPD patients than in HCs.^33^ However, the circulating level of ITIH4 in RA patients has not been examined yet. In this study, serum ITIH4 was decreased in RA patients than in HCs; meanwhile, ITIH4 disclosed good value to distinguish RA patients from HCs. Possible reasons might be that (1) ITIH4 inhibited neutrophilic migration and negatively correlated with the level of C5a, while large numbers of activated neutrophils and C5a were accumulated in RA patients; thereby, ITIH4 was declined in RA patients.^26^, ^34^, ^35^ (2) ITIH4 together with hyaluronan could form the covalent modification of hyaluronan with heavy chain (HA·HC) which suppressed inflammation and played crucial roles in RA etiology.^36^, ^37^, ^38^ Therefore, ITIH4 was correlated with disease risk of RA.

The inflammation‐regulating role of ITIH4 is conflicting; although some studies showed that ITIH4 served as a pro‐inflammatory cytokine, several evidence has disclosed the anti‐inflammatory properties of ITIH4 in COPD, inflammatory bowel disease, etc.^24^, ^39^, ^40^, ^41^ For instance, one previous study finds that ITIH4 is negatively related to IL‐6 in bronchoalveolar lavage fluid of COPD patients.^39^ Another study discovers that ITIH4 knockdown induces TNF‐α, IL‐1β, and IL‐6 expression in human placental choriocarcinoma cells.^40^ However, no clinical study explores the correlation of ITIH4 with inflammatory cytokines and disease activity of RA patients. In the current study, we found that ITIH4 was negatively linked with CRP, DAS28‐ESR, and pro‐inflammatory cytokines (including TNF‐α, IL‐6, and IL‐17A) in RA patients. The possible explanation was as follows: (1) The light chain of ITIH4 (also named bikunin) could suppress the activation of extracellular regulated protein kinase (ERK), while the latter one facilitated inflammatory response in RA.^42^, ^43^ Thus, ITIH4 was negatively linked with pro‐inflammatory cytokines in RA patients. (2) ITIH4 promoted the formation of HA·HC complexes, which inhibited TNF‐α activity via regulating tumor necrosis factor‐stimulated gene‐6 (TSG‐6) production.^36^, ^44^ Hence, ITIH4 was negatively correlated with TNF‐α in RA patients. (3) As described above, ITIH4 was negatively linked with IL‐6, IL‐17A, and TNF‐α, while the decline in those pro‐inflammatory cytokines was related to alleviated disease activity of RA patients.^45^ Hence, ITIH4 was negatively associated with some disease activity scores (including CRP and DAS28‐ESR) in RA patients.

Apart from the findings mentioned above, this study also investigated that ITIH4 was gradually increased in RA patients during the treatment; meanwhile, increased level of ITIH4 was related to better treatment outcomes in RA patients. The probable reasons could be that (1) as mentioned, ITIH4 was negatively correlated with inflammation in RA patients, whose inflammation level was declined after treatment.^46^ Hence, ITIH4 was gradually increased during the treatment of RA patients. (2) Elevated ITIH4 level represented alleviated inflammation level; meanwhile, the decline in inflammation level meant good treatment response and remission.^47^ Thus, increased ITIH4 was correlated with treatment response and remission in RA patients. (3) Increased ITIH4 level linked with decreased DAS28‐ESR whose decline correlated with high treatment response and remission rate.^48^ Therefore, elevated ITIH4 was related to treatment response and remission in RA patients.

Some limitations occurred in this study. First, the number of patients in the current study was relatively small; therefore, studies with a larger sample size to valid the findings were necessary. Second, this study enrolled HCs to evaluate the diagnostic value of ITIH4 for RA patients, while we did not recruit disease controls, which was required in the future studies. Third, the 12‐week follow‐up duration was relatively short; hence, a further study with longer follow‐up duration needed to be conducted. Fourth, the upstream pathway of ITIH4 was still unclear, which needed to be explored in the future study. Fifth, as mentioned above, citrullinated form of ITIH4 might be involved in the pathogenesis of RA, while we did not collect relevant data in the current study. Sixth, ITIH4 had been reported to regulate the expression of mannan‐binding lectin‐associated serine protease‐1 (MASP‐1), MASP‐2, and plasma kallikrein, which were key proteases for intravascular host defense, while the correlations of ITIH4 with MASP‐1, MASP‐2, and plasma kallikrein were unanswered and needed further study.^47^ Seventh, the underlying mechanism of how ITIH4 participated in the inflammation response of RA was not completely explored; hence, *in vivo* and *in vitro* studies were necessary.

In conclusion, we suggest that circulating ITIH4 correlates with disease risk, disease activity, and treatment outcomes of RA; consequently, it can be used as a potential biomarker which helps clinicians to stratify RA patients, individualize their treatment, and improve the outcomes in RA patients.

## CONFLICT OF INTEREST

The authors declare they have no conflict of interest.

## Supporting information

Fig S1

Table S1

## Data Availability

Data sharing is not applicable to this article as no datasets were generated or analyzed during the current study.
