# Supplementary figures and images for: Inter‐alpha‐trypsin inhibitor heavy chain 4: A serologic marker relating to disease risk, activity, and treatment outcomes of rheumatoid arthritis
Source: J Clin Lab Anal. 2022 Jan 22;36(3):e24231. doi: 10.1002/jcla.24231 (PMC8906037; doi:10.1002/jcla.24231)

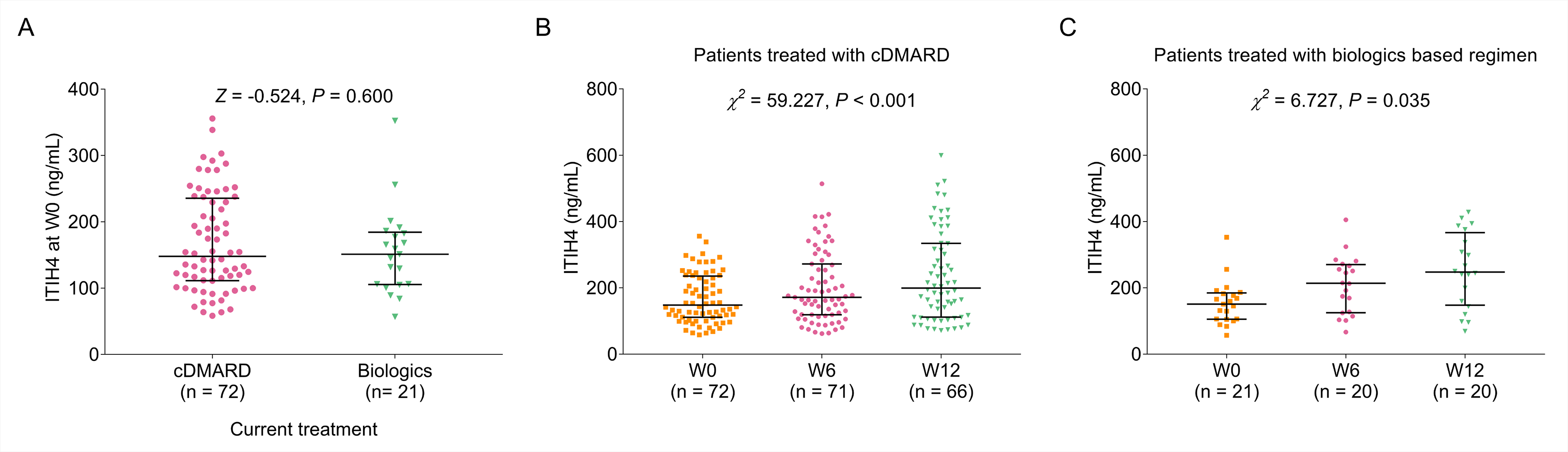

Supplement: Supplementary file 1 — Fig S1 [file JCLA-36-e24231-s001.tif]
