# Supplementary material for: Inter‐alpha‐trypsin inhibitor heavy chain 4: A serologic marker relating to disease risk, activity, and treatment outcomes of rheumatoid arthritis
Source: J Clin Lab Anal. 2022 Jan 22;36(3):e24231. doi: 10.1002/jcla.24231 (PMC8906037; doi:10.1002/jcla.24231)
Supplement: Supplementary file 2 — Table S1 [file JCLA-36-e24231-s002.docx]

Supplementary table 1. Factors affecting clinical response at W12 by logistic regression model analysis.

| Items | *P* value | OR | 95%CI | |
| --- | --- | --- | --- | --- |
|  |  |  | Lower | Upper |
| **Multivariate logistic regression** |  |  |  |  |
| Higher ITIH4 at W12 | 0.001 | 1.007 | 1.003 | 1.012 |
| Higher CRP | 0.005 | 1.032 | 1.009 | 1.055 |

W12, week 12; OR, odds ratio; CI, confidence interval; ITIH4, inter-alpha-trypsin inhibitor heavy chain 4; CRP, C-reactive protein.
